# The effects of FK1706 on nerve regeneration and bladder function recovery following an end-to-side neurorrhaphy in rats

**DOI:** 10.18632/oncotarget.21718

**Published:** 2017-10-10

**Authors:** Wansheng Gao, Xiangfei He, Yunlong Li, Jianguo Wen

**Affiliations:** ^1^ Department of Urology, The First Affiliated Hospital of Zhengzhou University, Zhengzhou, Henan Province, 450052, China

**Keywords:** FK1706, end-to-side neurorrhaphy, immunophilin ligands, neurogenic bladder, nerve regeneration

## Abstract

**Background:**

Immunophilin ligands are neuroregenerative agents binding to FK506 binding proteins, by which stimulate recovery of neurons in a variety of injury nerves. FK1706 is a novel immunophilin ligand which has neuroprotective and neuroregenerative effects but without immunosuppressive activity. At present, most reports about FK1706 in ameliorating nerve injury and functional recovery are limited to cavernous nerve injury and erectile function recovery. This study aimed to demonstrate the effects of FK1706 on nerve regeneration and bladder function recovery following an end-to-side neurorrhaphy in rat models.

**Method:**

The numbers of regenerated myelinated axons of the pelvic parasympathetic nerve (PPN) in the three groups’ rats (FK1706 + ETS, ETS and control groups) were evaluated. Their intravesical pressure (IVP), S100β and growth associated protein 43 (GAP43) expressions were also compared.

**Results:**

In FK1706 + ETS group, 90% the rats showed that the frequency of FG labeled neurons was larger than the 3.5 cutoff value, 100% the rats showed that the frequency of FG-FB double-labeled neurons was larger than the 5.5 cutoff value. The average maximum of IVP in FK1706 + ETS group reached 76.3% of the value in control group. Their average number of myelinated axons of regenerated PPN reached 80% of the amount in control group. The nerve regeneration-associated markers data indicated that the expression level of S100β and GAP43 in FK1706 + ETS group was approximately 2-fold higher than that of ETS group (P < 0.05).

**Conclusions:**

After end-to-side neurorrhaphy, FK1706 effectively enhanced the nerve regeneration and bladder function recovery.

## INTRODUCTION

Neurogenic bladder because of all kinds of neural diseases such as neural tube defects, spinal cord injury, diabetes and peripheral nerve trauma, is a major medical problem affecting overall health for these patients. Approximately 10% of these patients eventually died from renal failure caused by neurogenic bladder dysfunction (NBD) each year [1]. NBD has been treated by intradural End-to-side (ETS) neurorrhaphy of lumbar and sacral nerve, ameliorating urination dysfunction and hydronephrosis for these patients [2]. ETS neurorrhaphy is a nerve rerouting surgery which sutures the distal stump of an injured nerve to the side of an adjacent healthy nerve to promote nerve regeneration and functional reconstruction with preserving adjacent nerve function simultaneously [3]. One important advantage of ETS neurorrhaphy is the donor nerve function could be preserved well. However, the results of nerve repair and bladder function reconstruction remain unsatisfactory after ETS neurorrhaphy since the donor nerve is intact without any injury which reduces the effect of axonal regrowth. Our previous study indicated that after ETS neurorrhaphy, the number of regenerated axons and intravesical pressure (IVP) only reached half of the normal value [4]. How to further improve the nerve regeneration and bladder function recovery is a challenge for urologists and neurosurgeons at present.

Immunophilin ligands represent a new class of therapeutic agents with neuroprotective and neuroregenerative properties [5]. The immunosuppressant ligand tacrolimus, that is FK506, was found to have neuroprotective and neurotrophic effects including stimulation of axonal regeneration and enhancement of functional recovery in a variety of neurodegenerative disease models such as spinal cord injury, stroke and erectile dysfunction [6-11]. However, the immunosuppressive activity of FK506 limits its long term use in patients for nerve repair and functional recovery. FK1706, a derivative of FK506, is a novel immunophilin ligand which has neuroprotective and neuroregenerative effects but without immunosuppressive activity [12, 13]. At present, most reports about FK1706 in ameliorating nerve injury and functional recovery are limited to cavernous nerve injury and erectile function recovery [14-17]. The results of nerve regeneration and bladder function recovery of the FK1706 after ETS neurorrhaphy are still not being determined. This study is to explore whether FK1706 could enhance the recovery of injured nerve and bladder function after an ETS neurorrhaphy of autonomic nerve and somatic nerve in the peripheral nervous system.

## RESULTS

### Retrograde nerve tracing

**Figure 1:**
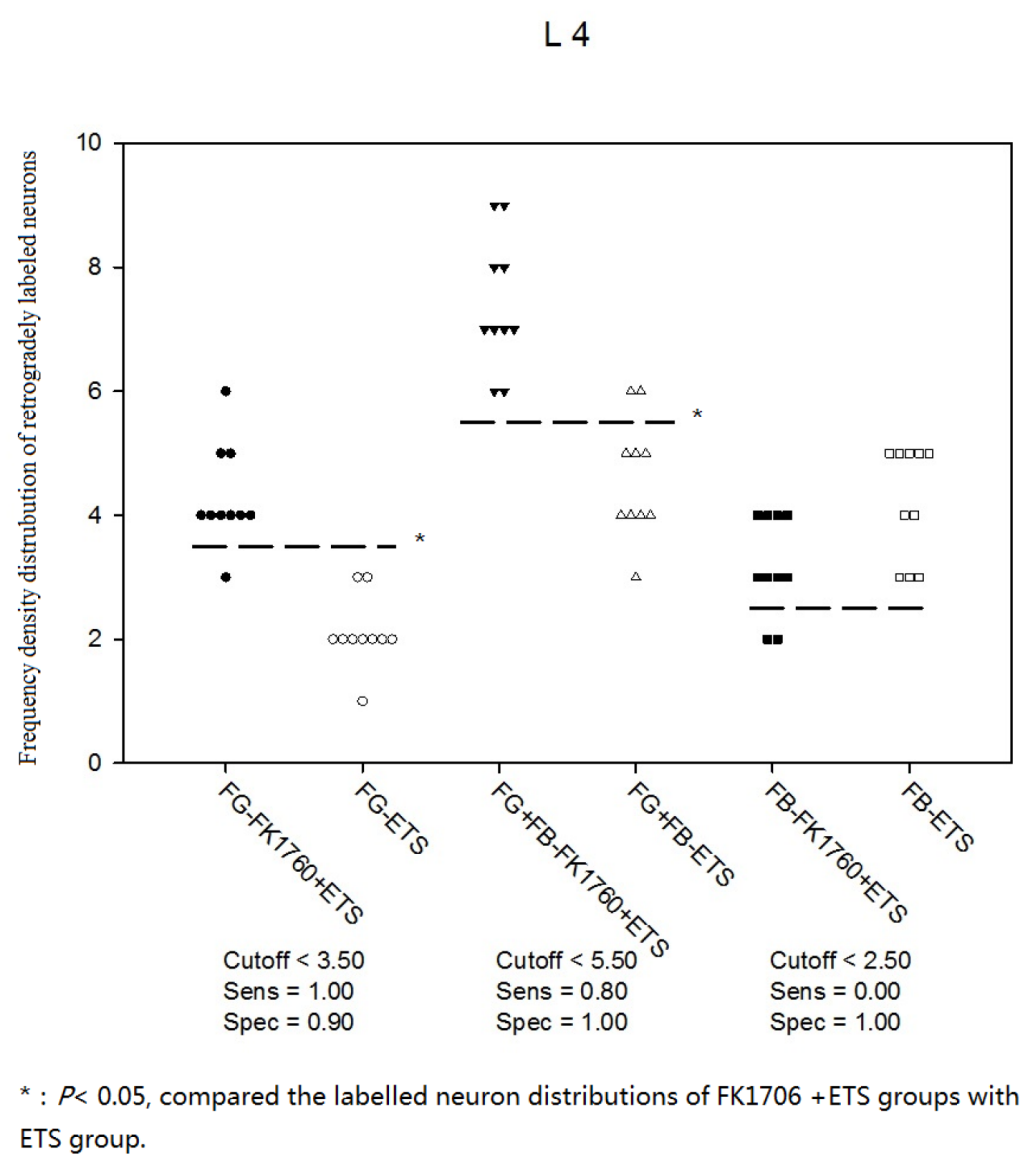
Frequency density distribution of neurons retrogradely labeled with FG, FG-FB and FB

**Figure 2:**
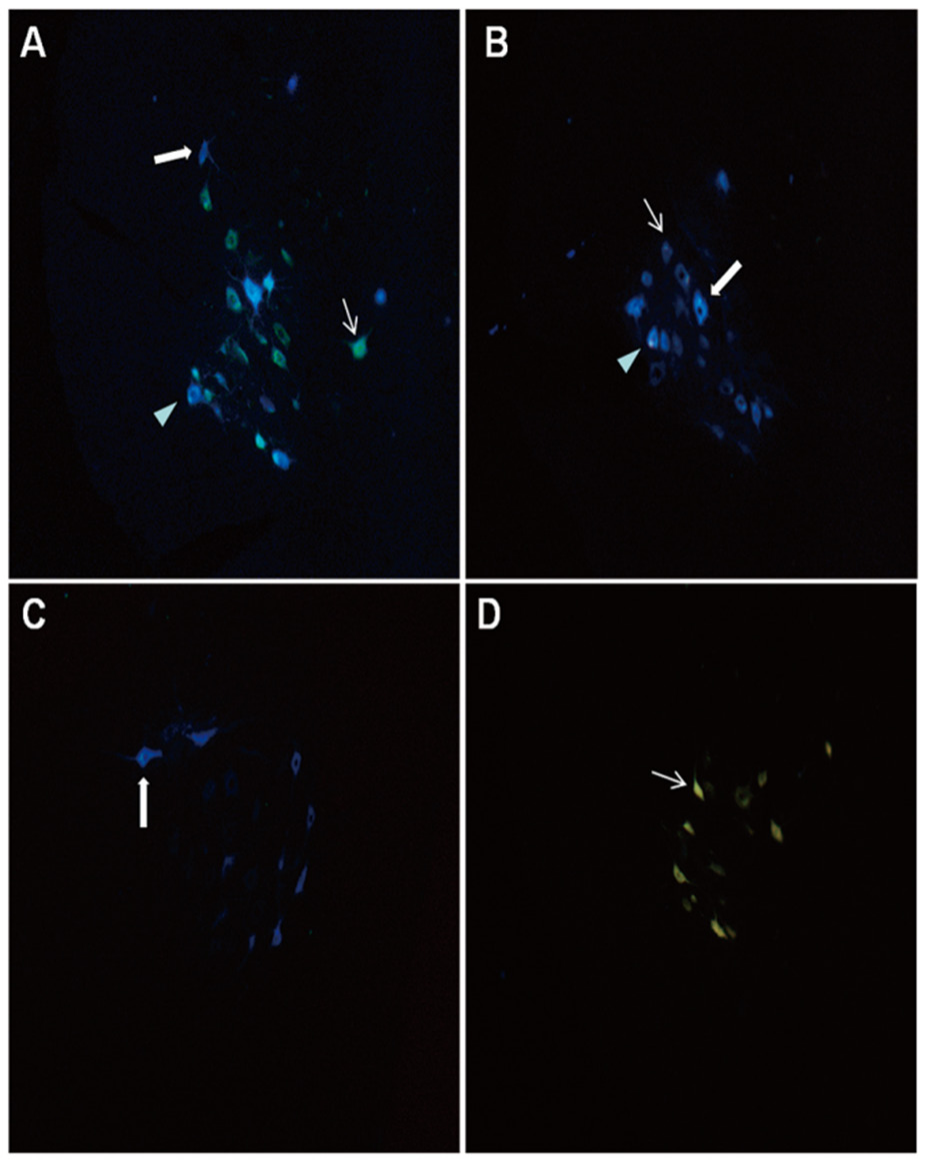
Distribution of retrogradely labeled neurons observed under ultraviolet fluorescence microscope: → FG labeled neurons; ➡ FB labeled neurons; ► FG-FB double labeled neurons **(A)** Neuron distribution on L4 segment in FK1706 + ETS group; **(B)** Neuron distribution on L4 segment in ETS group; **(C)** Neuron distribution on the L6 segment in control group, **(D)** Neuron distribution on L3-L5 segments in control group.

The Frequency density distribution of retrogradely labeled neurons based on ROC analysis indicated that FG single-labeled neurons and FG-FB double-labeled neurons were mainly located in L4 (Figure 1). FB single-labeled neurons were only detected in L3-L5 of FK1706 + ETS and ETS groups (Figure 2A and 2B). The cutoff value of FG labeled and FG-FB double-labeled neurons in FK1706 + ETS group were < 3.5 and < 5.5, respectively, significantly differed from ETS group, suggesting FK 1706 significantly enhanced the neuron regeneration. The labelling sensitivity of FG only is better than that of FG-FB, but its specificity of is not better than double labelling. FB labeled neurons has no sensitivity (P < 0.05; Figure 1). There was no labeled neuron noted in left L6 segment in FK1706 + ETS and ETS groups. In the control, FG single-labeled and FB single-labeled neurons were observed in the segments of L3-L5 and L6, respectively, and there were FG-FB double-labeled neurons found. No labeled neurons were detected on the contralateral side in the spinal cord (Figure 2C and 2D).

### Evaluation of bladder function recovery

**Figure 3:**
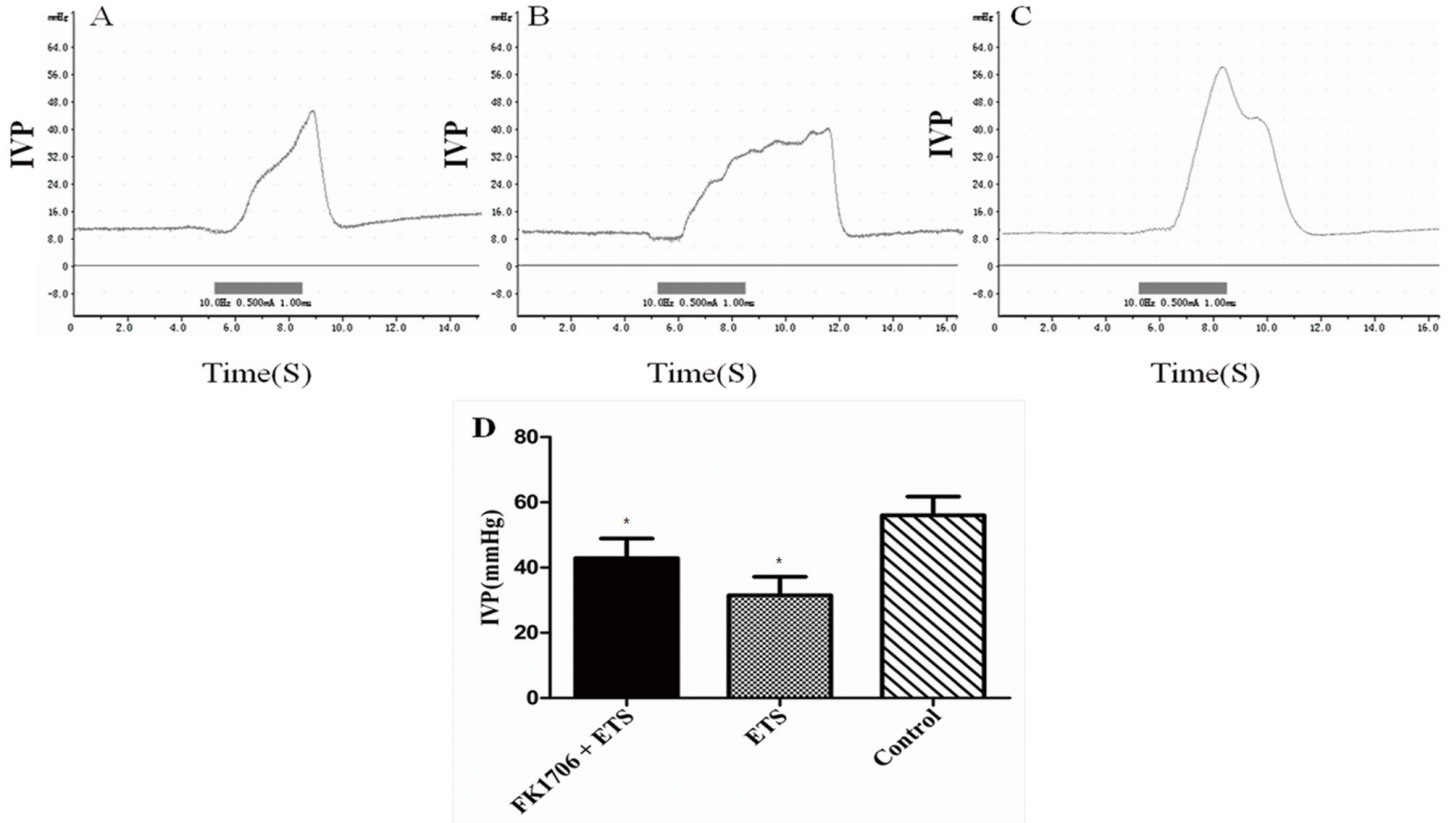
Cystometric recordings of the intravesical pressure Flow rate of bladder perfusion (0.9% saline solution) was 2.5 ml/h (at room temperature). **(A)** intravesical pressure (IVP) of FK 1706 + ETS group; **(B)** IVP of ETS group; **(C)** IVP of the control group; **(D)** IVP comparison among the three groups: The IVP value of FK1706+ETS group was significant higher than that of ETS group (^*^: P<0.05).

**Figure 4:**
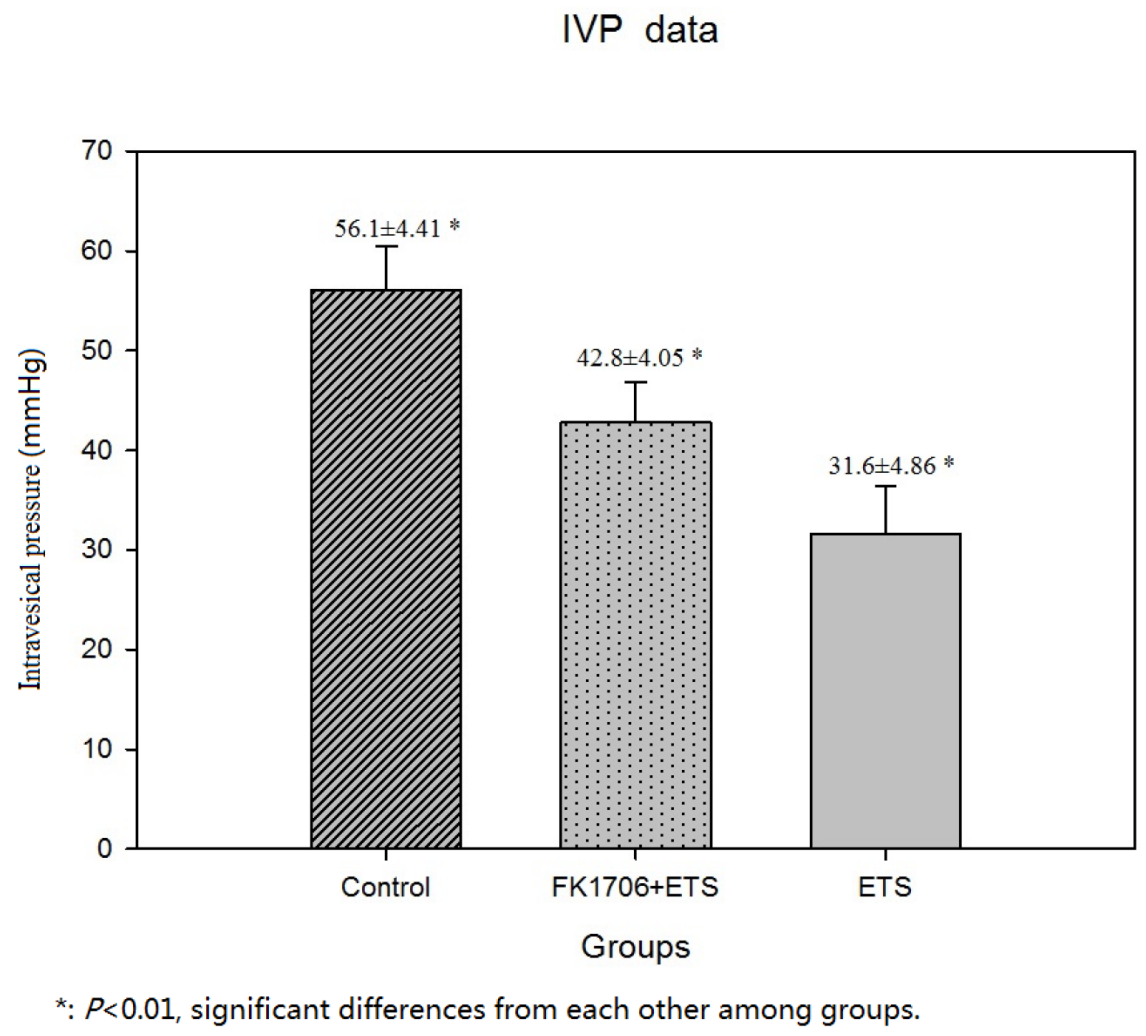
Postoperative IVP measurement

The effects of FK1706 on bladder function recovery in rats were evaluated by IVP, which correlates detrusor contraction in urinary dynamics examination, mainly representing bladder function. The IVP had an obvious rise during electrostimulation in FK1706 + ETS and ETS group (Figure 3A, 3B and 3C). The mean IVP peak in FK1706 + ETS group was significantly higher than that of ETS group. The IVP peak elicited by the artificial established neural reflex pathway was 42.8 ± 4.05 mmHg (FK1706 + ETS) and 31.6 ± 4.86 mmHg (ETS), respectively. There was a significant difference of the mean IVP peak between the FK1706 + ETS group and ETS group (*P* < 0.05). For control group, when the left L6VR was stimulated using the same stimulating parameters (Figure 4), the mean IVP peak was 56.1 ± 4.41 mmHg (Figure 3D), and when the left corresponding L4VR was stimulated using the same parameters, IVP did not change.

### Morphological examination and myelinated axons counting of regenerated PPNs

**Figure 5:**
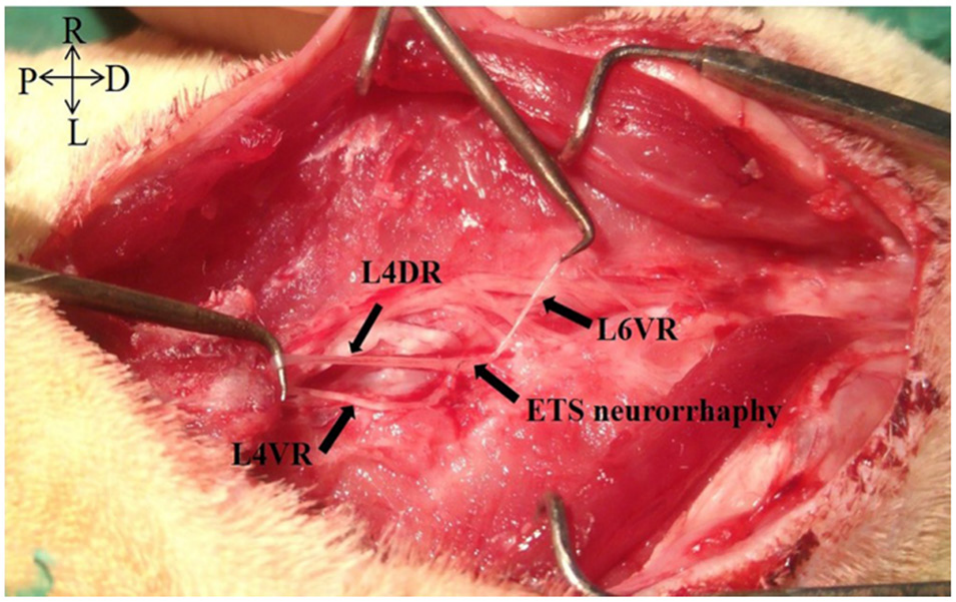
The macroscopic photo of L4VR-ETS Neurorrhaphy at 4 months after surgery

**Figure 6:**
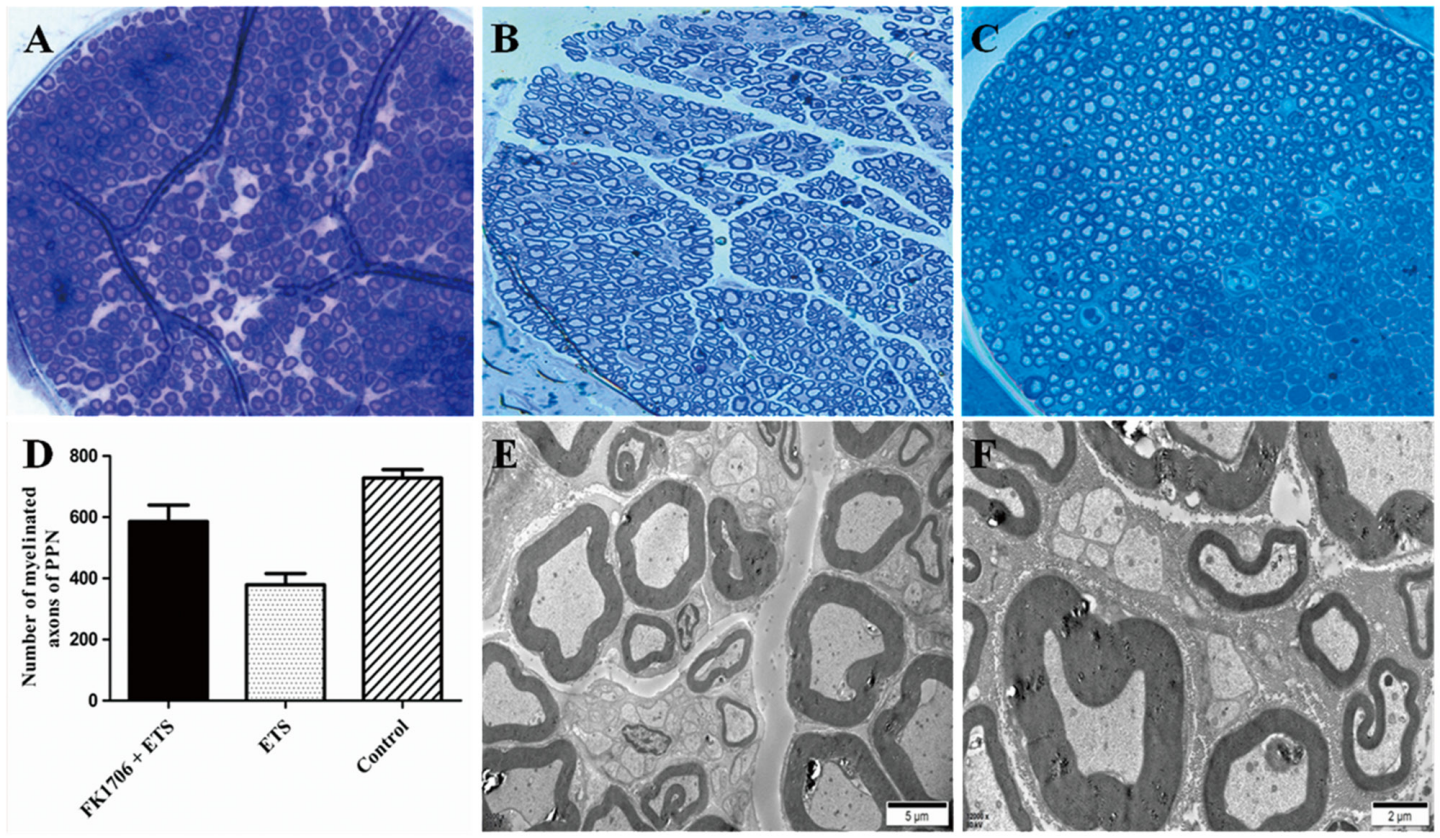
The photomicrograph of semithin sections of pelvic parasympathetic nerves (PPNs) **(A)** Axons regrowth in the FK1706 + ETS group; **(B)** Axons regrowth in the ETS group; **(C)** Axons was distributed uniformly in control group; **(D)** Histogram of myelinated axons of pelvic parasympathetic nerve (PPN) in the three groups; **(E)** Ultrastructure of regenerated PPNs showing no Wallerian degeneration in ETS group under transmission electron microscopy; **(F)** Ultrastructure of regenerated PPNs showing no Wallerian degeneration in FK1706 + ETS group under transmission electron microscopy.

Figure 5 was clear shown that the neurorrhaphy site of the distal end of left L6VR and the lateral face of the left L4VR already grew tightly 4 months after surgery. The macroscopic photo illustrated that newborn axons could crossed the neurorrhaphy site into the receptor autonomic nerve from intact somatic nerve. The average number of myelinated axons of the preganglionic PPN was 585.6 ± 53.8 in FK1706 + ETS group, 378.9 ± 36.7 in ETS, and 728.3 ± 27.6 in control group. The photomicrograph observation of semithin sections of PPNs in the FK1706 + ETS group and ETS group both showed the distinct axons regrowth (Figure 6A and 6B). The distribution and morphology of axons regrowth were more uniform in the FK1706 + ETS group, which was close to control group (Figure 6C). There was a significant difference in the number of myelinated axons of PPN between FK1706 + ETS group and ETS group (*P* < 0.05, Figure 6D). No myelin swelling or degeneration was observed in the semithin sections of regenerated PPN. The ultrastructure of regenerated PPNs indicated that a large number of myelinated axons and unmyelinated axons existed and no Wallerian degeneration occurred in FK1706 + ETS and ETS groups (Figure 6E). Additionally, more myelinated axons could be observed (Figure 6F).

### Expression of axonal regrowth associated protein by Western blotting

**Figure 7:**
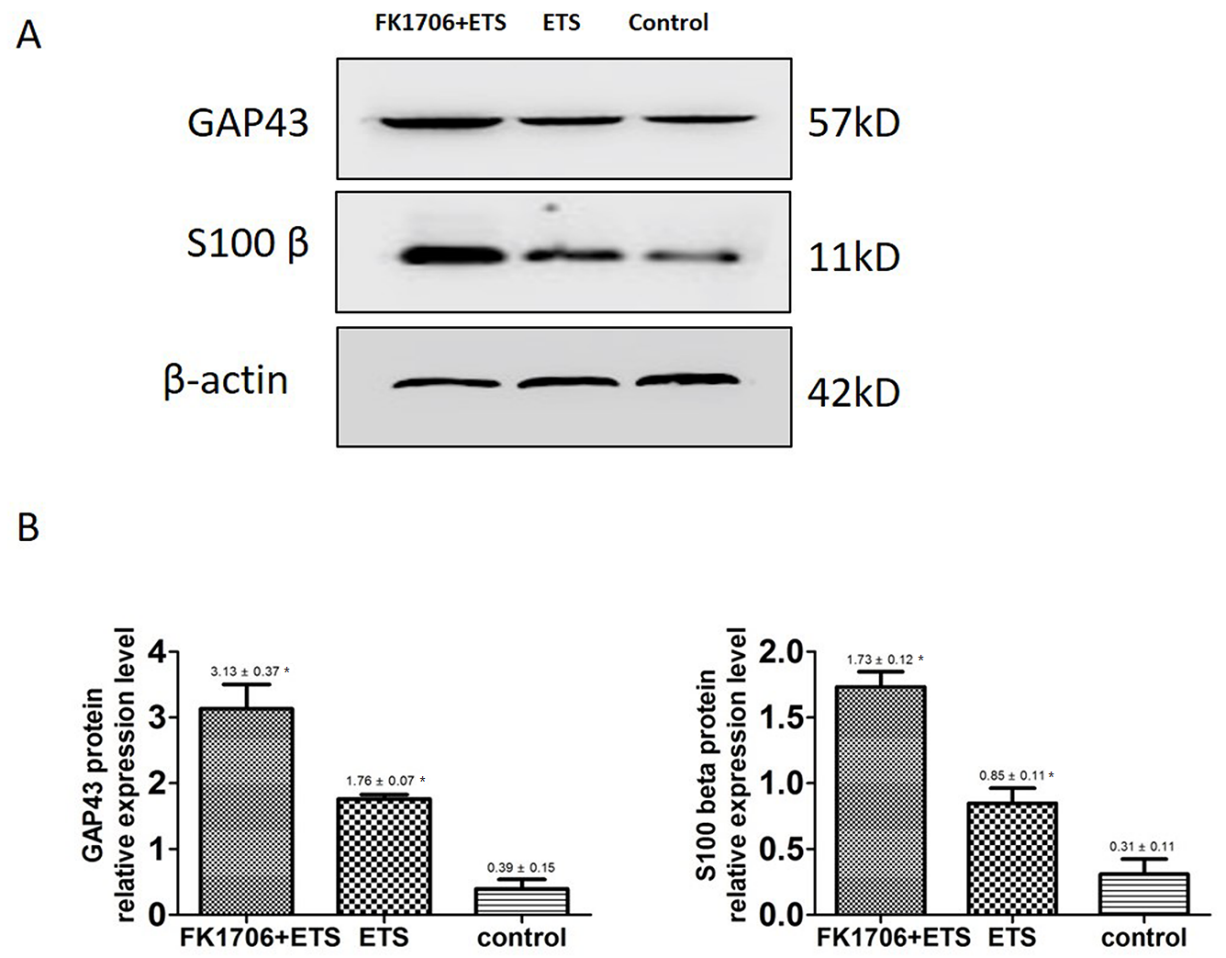
**(A)** Western blotting images of S100β and GAP43 expression. **(B)** The histograms showed the relative expression levels of S100β and GAP43 (^*^: P<0.05 between FK 1706 + ETS and ETS).

Although the above illustrated the differences of nerve regeneration and functional recovery between FK1706 + ETS group and ETS group by means of morphological and functional examination, it could not discern the nerve regeneration associated protein expression differences between different groups. Therefore, the expressions of S100β and GAP43, two nerve regeneration-associated markers, in the regenerated autonomic nerve distal to neurorrhaphy site were investigated on the 4th months after ETS neurorrhaphy. The expression level of S100β in the regenerated receptor nerve in FK1706 + ETS group was 2.04 times higher that of ETS group (Figure 7, *P* < 0.05). The S100β concentration in ETS group was similar to the control and there was no significant difference between them (*P* > 0.05). The GAP43 expression level in FK1706 + ETS group was 1.78 times higher than that of ETS group (*P* < 0.05) and There was no significant difference in the expression level of GAP43 between ETS and control groups (*P* > 0.05). Western blotting data of axonal regrowth markers were further confirmed the morphological and functional examination results of them.

## DISCUSSION

ETS neurorrhaphy has been successfully used to treat voiding dysfunction with neurogenic bladder owing to various neurogenic diseases and nerve injury, which brought a great hope for these patients. This technique has significant advantages over traditional reconstructive methods in the peripheral nervous system. Previously we have reported that nerve regeneration and functional recovery could be achieved through ETS neurorrhaphy with preserving the donor nerve function simultaneously. The main regeneration mechanism was that the somatic motor axons grown into autonomic nerve was through axon collateral sprouting only [4, 18]. However, the results of nerve regeneration and function rehabilitation after ETS neurorrhaphy were unsatisfactory, which is a key point to the application of this technique in clinic in the future. Therefore, ETS neurorrhaphy in combination with some nerve growth factors to enhance the effects of nerve regeneration and functional rehabilitation was investigated. When an end-organ becomes denervated, reinnervation can occur in following two ways: through collateral branching of intact axons or by regeneration of the injured axon [19]. If 20-30% of the axons are damaged, collateral branching is the major mechanism of recovery, it begins in the first 4 days following injury and will continue for about 3-6 months, until recovery occurs [20, 21]. There are more axonal branches that sprout than the actual number of nerves that end up eventually innervating a target-organ [22]. Those branches that do not receive neurotrophic factors from the target-end organ undergo a pruning process and are destined to degenerate [23, 24]. If > 90% of the axon population within a nerve damaged, axonal regeneration is the primary means for recovery [25].

FK1706 derived from FK506 is a novel non-immunosuppressive immunophilin ligand with neuroregenerative and neurotrophic characteristics, including stimulation of axonal regeneration and enhancement of functional recovery in a variety of neurodegenerative animal models [26, 27, 28]. While no changes occurred after 1 week of commencing of FK1706 administration (2 weeks after streptozotocin injection), changes in expression more than 1.5-fold were observed for fast-type muscle genes such as troponin C type 2 (*TNNC2*) and *MYLPF* at two weeks of FK1706 administration (3 weeks after streptozotocin injection). Daily oral administration of FK1706 improved mechanical allodynia via Peptidyl-prolyl cis-trans isomerase (FKBP4) and the Ras protein / Raf kinase/mitogen-activated protein kinase (Ras/Raf/MAPK) signaling pathway. It also improved mechanical allodynia associated with the recovery of intraepidermal nerve fiber density in a painful diabetic neuropathy in rats [29]. Bella et al. have reported that FK1706 could enhance erectile function recovery in bilateral cavernous nerves crush injury rat model, indicating that it is a potential therapeutic agent for neurodegenerative diseases [5]. The neurotrophic effects of FK1706 show a significant clinical value for nerve regeneration and functional rehabilitation under some circumstances. However, after ETS neurorrhaphy between autonomic and somatic nerve in the peripheral nervous system, the results of nerve regeneration and bladder function recovery of using FK1706 (FK1706 in combination with ETS) remain uncertain so far.

This study investigated the effect of FK1706 in combination with ETS on nerve regeneration and bladder function recovery. The nerve regeneration and bladder function rehabilitation were evaluated by morphological examination, nerve regeneration associated protein measurement and IVP measurement. Yamaji et al. have proved that motor function could be improved in FK1706 treated rats after spinal cord injury, which was consistent with morphological changes [17]. Hayashi and Bella both reported that FK1706 is effective in recovery of erections after cavernous nerve injury in rat models, which was dependent with dose [5, 12]. We selected the moderate dosage as the experimental dose in FK1706 + ETS group in the initial study.

In the retrograde nerve tracing study, the appearance of retrogradely labeled neurons implied that the artificial neural pathway had been successfully established between the recipient nerve and the donor nerve. The numbers of labeled neurons indicated the regenerated nerve fibers numbers to some extent. The appearance of FG single labeled neurons and FG-FB double labeled neurons implied that the nerve regeneration mechanism was axonal collateral sprouting. At 4 months after surgery, we performed density-frequency analysis of labelling neurons on L4 and found there were significant changes in the regenerated myelinated axons after FK1706 application. The numbers of FG single-labeled neurons and FG-FB double-labeled neurons detected in FK1706 + ETS group both were more than those in ETS group, which were consistent with the results of myelinated axons counting. It could be observed that there were FB single labeled neurons in the L4, but there was no statistical difference (Figure 1). The average number of regenerated myelinated axons in preganglionic PPN distal to coaptation in FK1706 + ETS group was obvious more than that in ETS group, which indicated that more regenerated myelinated axons could reach bladder through the coaptation site after FK1706 treatment. We previously reported that the number of myelinated axons of regenerated PPN after ETS neurorrhaphy could reach 54.8% of normal amount of PPN in control group. In this study, the average number of myelinated axons of regenerated PPN in FK1706 treated group after ETS neurorrhaphy could reach 80% of normal amount in control group, this result was an encouraging. Both retrograde nerve tracing and myelinated axons counting demonstrated that more myelinated axons crossed the coaptation site into the receptor nerve and the nerve regeneration was enhanced by using FK1706 after neurorrhaphy.

For bladder functional recovery, the IVP curve indicated that bladder reinnervation could be achieved and regulated better in FK1706 + ETS group. The IVP elevation and urination was observed when the left L4VR proximal to the coaptation was stimulated. The average maximum of IVP in FK1706 + ETS group was significant higher than that in ETS group, which could reach 76.3% of normal pressure. The IVP revealed a quantified index of bladder function. IVP change demonstrated that bladder functional recovery could be improved by using FK1706 after ETS neurorrhaphy, which was consistent with the results of nerve regeneration and protein expression in this study.

After the neurorrhaphy, functional MRI examination found that the brain stem can form a new urinary control center [30]. Through the neurorrhaphy between control of urinary nerve and donor nerve, donor nerve axons re-growth into the recipient nerve, with the brain - urinary central reconstruction, the new nerve reflex pathway is established, the electrical stimulation of donor nerve L4VR can achieve urinary reflex. This effect has been proven by retrograde nerve tracing and bladder pressure changes. In the FK1706 group, the ETS group, and the control group, their residual urine volume were: 0.05 ± 0.02 ml, 1.16 ± 0.24 ml, 2.36 ± 0.34 ml (P <0.001for each group).

Axonal regrowth was induced and enhanced by FK1706 after nerve injury. S100β and GAP43 are the usual protein markers associated with axonal growth and regeneration. Their expression levels in the regenerated PPNs after neurorrhaphy were higher than in control group. After neurorrhaphy, these two protein expressions was significantly increased following FK1706 application, these changes were also supported by morphological examination, suggesting that FK1706 had a therapeutic potential for nerve repair in the peripheral nervous system to aid in improve functional recovery after nerve injury. Since the neurotrophic effects of FK1706, via FK binding protein and activation of Ras/Raf/MAPK signaling pathway, induce nerve growth factors mediated axonal regrowth [13, 29, 31, 32]. For axonal regrowth, FK1706 was more effective than FK506 and it did not inhibit interleukin-2 production or lymphocyte proliferation (non-immunosuppressive immunophilin ligand) [13]. Compared with the data from our previous study and with the ETS group, we demonstrated that after FK1706 administration, the IVP was significantly increased (Figure 3D); the nerve regeneration indicated by S100β and GAP43 was also significantly improved (Figure 7). These demonstrated that FK1706 significantly enhanced the bladder function recovery due to the better nerve regeneration. If FK1706 was used 1 week after nerve injury, it was still effective, indicating that FK1706 has a reasonable therapeutic time-window [17]. This characteristic would be more beneficial in some acute clinical circumstance, since the initial clinic focus is usually on controlling bleeding, edema and inflammation induced by injury.

For neuroprotective and nerve regenerative therapy, FK506 and Rapamycin have potential advantages over conventional nerve growth factors since they are non-peptides, lipophilic and selectively effective for damaged nerves. When long term use, their adverse effects such as immunosuppression and nephrotoxicity have been clinically concerned. To circumvent these problems, non-immunosuppressive analogs of FK, GPI-1046 and FK1706 with neurotrophic and neuroprotective effects have been developed. These non-immunosuppressive analogs have been verified to preserve cavernous tissue structure and erectile function in rats after CN injury [5, 12, 33]. Therefore, FK1706, the novel potential therapeutic agent will be likely to be applied for nerve repair and neurogenic bladder treatment in the future.

## MATERIALS AND METHODS

Briefly, retrograde nerve tracing was performed by retrograde fluorescent tracers: Fluorogold (FG, Biotium Inc., Hayward, CA) and Fast Blue (FB, Polysciences Inc., Warrington, PA) to determine the effect and origin of the axonal regrowth after ETS neurorrhaphy. The postoperative IVP measurement was carried out to evaluate the bladder function recovery. The status of regenerated pelvic parasympathetic nerve (PPN) was assessed by morphological examination and Western blotting.

### Surgical procedure

All the experiments concerning animals in this study were approved by the First Affiliated Hospital Medical Ethics Committee of Zhengzhou University and the animals were treated according to National Institutes of Health guide for the care and use of Laboratory animals. In this study, 30 adult male Sprague-Dawley rats that weighed between 220 and 280g provided by the experimental animal center of Zhengzhou University were used. All efforts were made to minimize the number of rats used and their suffering. The rats were randomly divided into 3 groups of 10 each.

**Figure 8:**
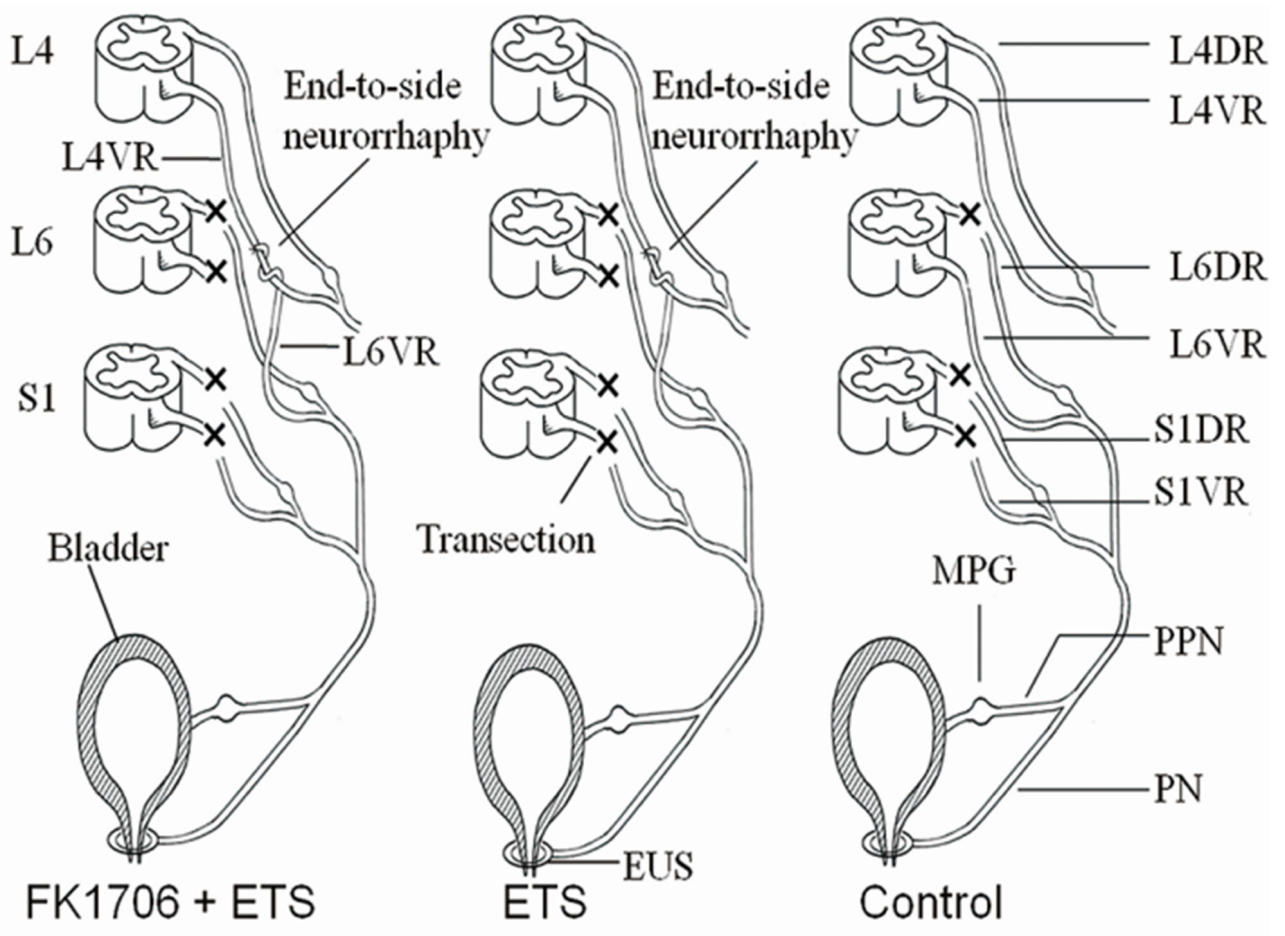
Schematic illustration of the surgical procedures

**Figure 9:**
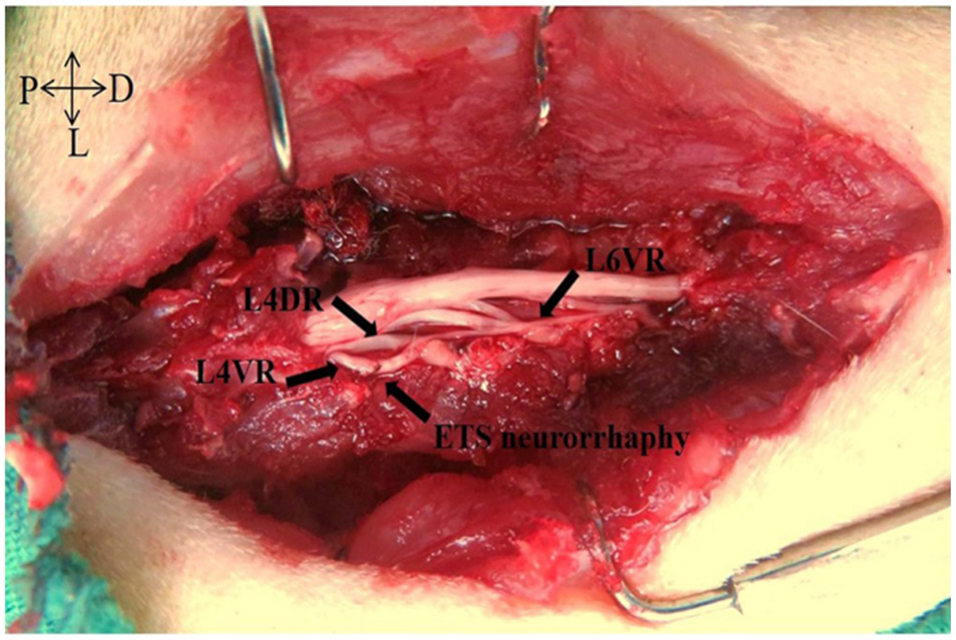
L4VR-ETS Neurorrhaphy

All the surgical was performed with the rats under deep anesthesia through intraperitoneal injection using 10% chloral hydrate (300 mg/kg, Sinopharm Chemical Reagent, Shanghai, China). A schematic illustration shows the different surgical procedures performed within the 3 groups and the entire surgical procedures were performed by the same surgeon under aseptic conditions (Figure 8). In FK1706 + ETS group, the rats were subjected to laminectomy from L3 to S1 vertebra on the dorsal median incision to expose the spinal nerves with lying prone. The ventral roots (VRs) and dorsal roots (DRs) from L4 to S1 were exposed in the dura. The L6VR, L6DR, S1VR and S1DR on the left side were transected at L4 level. The distal stump of the left L6VR (recipient nerve) was wrapped in a helicoid fashion around the longitudinal axis of the left L4VR (donor nerve) and then the distal stump was sutured to lateral face of the intact L4VR in an ETS fashion with 10-0 nylon sutures (Ethicon, Shanghai, China) under a surgical microscope with 12.5× magnification (OPMI 9, Carl Zeiss, Germany) (Figure 9). L4 is a somatic nerve involved in the composition of the sciatic nerve; it provides donor nerve as the main source of nerve regeneration. The proximal stumps of left L6VR and S1VR were ligated and buried into the adjacent muscle to prevent spontaneous nerve regeneration into the distal stumps. The muscles and fascia were closed with absorbable 4-0 stitches in the layers.

**Figure 10:**
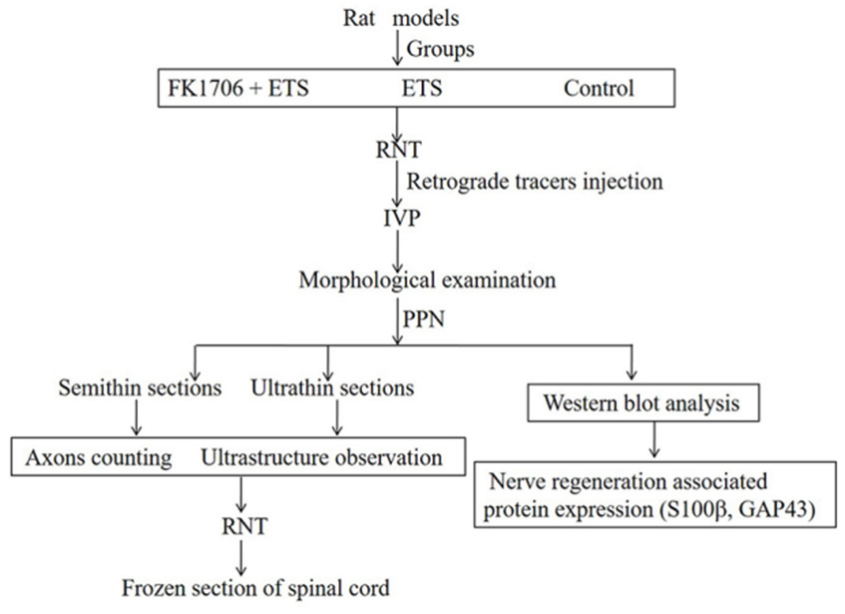
Experimental design and process overview

FK1706 was synthesized and purified in Shanghai Simr Biotech Co., Ltd. (Simr, Shanghai, China). FK1706 was prepared with 4% HCO-60/ETOH in distilled water. A subcutaneous injection of Medium dose of FK1706 (0.3 mg/kg) was continued 5 days per week for 8 weeks for all the rats in this group after surgery. In ETS group, the same surgical procedures as FK1706 + ETS group were performed but without FK1706 treatment after surgery. In control group, all the VRs and DRs from L6 to S1 on the left side were exposed and transected at L4 level but the L6VR was preserved as normal control. In ETS group and control group, the same dose of 4% HCO-60/ETOH in distilled water was given by the same injection method. Penicillin (0.2 million U/day) was administrated intraperitoneally for 3 consecutive days after surgery. The rats were caged separately with free access to water and standard food at suitable temperature with normal light dark cycle. Four months later, myelinated axons counting and IVP were measured to investigate efficacy of nerve regeneration and bladder function recovery between FK1706 + ETS group and ETS group. Western blotting was performed to analyze expression level of axonal regrowth associated protein in different groups. More details about the entire experimental procedures for the rats are shown in Figure 10.

### Retrograde nerve tracing

**Figure 11:**
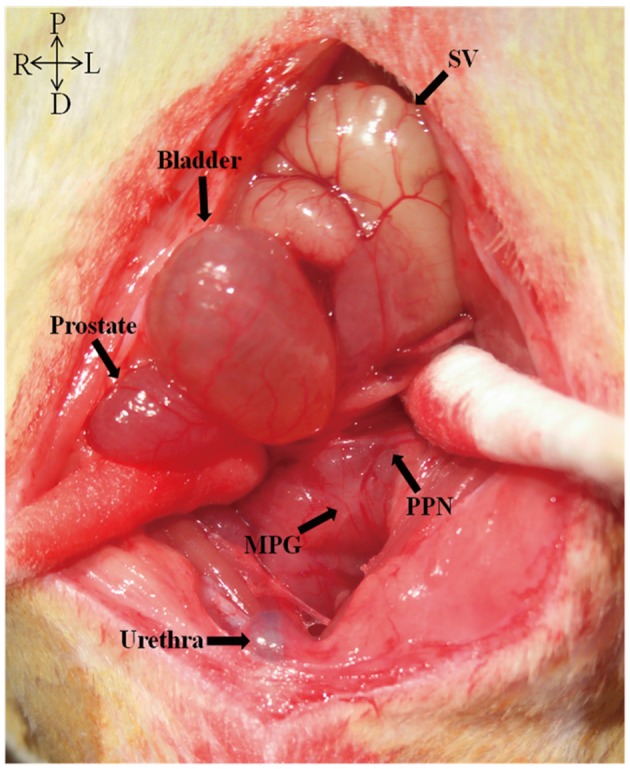
Left major pelvic ganglion (MPG) and the pelvic parasympathetic nerve (PPN)

Four months after surgery, the rats were re-anesthetized for retrograde nerve tracing. FG was dissolved in distilled water to make a 4% suspension and stored at 4°C in the dark. The left major pelvic ganglion (MPG) where the pelvic PPN terminates was exposed (Figure 11) and 0.5μl 4% FG was injected into the left MPG via a 1-μl micro-syringe. To avoid leakage of the FG and contamination of adjacent tissues, the injection site was covered with glycerin. Three days after the injection of the FG, the rats were re-anesthetized and 1μl 3% FB in the same way was injected into the left sciatic nerve (SN), that is the peripheral nerve component of L4VR. After allowing 7 days of retrograde transport, IVP measurement was performed under conscious condition. Then the left preganglionic PPN composed of L6 and S1 distal to ETS neurorrhaphy site were harvested for morphological examination and Western blotting analysis before perfusion. Afterwards the thoracic cavity was opened and the heart was quickly dissected under deep anesthesia: a cannula was inserted into the left ventricle and ascending aorta via a small incision of apex cordis. 200 ml of 0.9% saline was perfused into the left ventricle as soon as possible followed by 500 ml cold 0.1 M phosphate buffer saline (pH 7.4, 4°C) containing 4% paraformaldehyde. After perfusion, the spinal cord segments from L3-S1 were harvested and post-fixed in 4% paraformaldehyde at 4°C for 4h. Then they were embedded and frozen in Tissue-Tek (Tissue-Tek, Sakura Finetek, Torrance, CA) following a dehydration by 30% sucrose solution overnight at 4°C. A serial of 20 μm thick transverse sections of the spinal cord were cut using a cryostat microtome (CM1950, Leica, Germany) and mounted on gelatinized slides. Every 4th section was examined. Retrograde labeled neurons were counted by ultraviolet fluorescence microscope (DM4000, B LED, Leica Microsystems Ltd., Germany) equipped with a CCD camera (DFC450 C, Leica Microsystems Ltd., Germany).

### Postoperative IVP measurement

On the 7th day of after injecting the retrograde tracer FB, the rats were re-anesthetized and a lengthwise small incision in the abdomen was made to identify the bladder. A 5F polyethylene catheter was inserted into the dome of the bladder and was fixed appropriately with a purse-string suture. The catheter was tunneled subcutaneously and anchored to the skin on the back with suture so that the catheter could not be manipulated by the rats. Then, the abdomen was closed and the back incision was reopened with the rats lying prone to examine the neurorrhaphy site. The catheter was connected to a micro-infusion pump via a T-shape tube, and a pressure transducer with a computer, which is a biological signal converter system (BL-420, Chengdu TME Technology Co., Ltd., Chengdu, China) to record the increased bladder pressure (mmHg). The bladder was continuously infused with saline at room temperature through the micro-infusion pump at the rate of 2.5ml/h. A bipolar hook electrode was placed on the left L4VR proximal to the neurorrhaphy site in FK1706 + ETS group and ETS group. Electric stimulation was a square pulse and transmitted at a frequency of 10 Hz for 1 ms with an intensity of 0.5 mA. The left L4VR was repeatedly stimulated 3 times by the same parameters. When urination, the average maximum pressure of each rat in FK1706 + ETS group and ETS group were calculated for statistical analysis. For control group, the left corresponding L6VR was stimulated using the same parameters and the average maximum pressure when urination was calculated as normal control value.

### Morphological observation and myelinated axons counting of regenerated pelvic parasympathetic nerves

After IVP measurement, morphological observation of the regenerated PPN was performed in FK1706 + ETS and ETS group. The left regenerated preganglionic pelvic parasympathetic nerves (PPNs) distal to neurorrhaphy site were harvested under surgical microscope without perfusion after examination of the neurorrhaphy site. The obtained PPNs were randomly divided into two groups for morphological examination and Western blotting. For morphological examination, specimens were fixed in cacodylate-buffered 2.5% glutaraldehyde overnight and post-fixed in a 1% osmium tetroxide for 24h in 0.1 M sodium cacodylate buffer, dehydrated through a graded ethanol series and embedded in resin. The embedded nerve specimens were cut into 1 μm thick transverse sections using an ultramicrotome (EMUC7, Leica, Germany), stained with 1% toluidine blue (Sigma-Aldrich, Germany) for microscopic observation. The regenerated myelinated axons number of PPNs was calculated with the aid of automated image analysis software, Image-Pro plus 6.0 for windows (Media Cybernetics, Rockville, MD). After observation of the regenerated myelinated axons of the preganglionic PPNs, the embedded nerve specimens were further cut into ultrathin sections, stained with uranyl acetate and lead citrate to observe ultrastructure by transmission electron microscope (JEM-1400, Japan). For control group, left corresponding preganglionic PPNs were harvested and performed referring to the above procedures.

### Western blotting analysis

Western blotting analysis of regenerated PPN was performed to further evaluate the nerve regeneration on molecular level. The randomly selected other PPNs were used to analyze axonal regrowth associated protein expression level by Simple Western assays [34, 35]. S100β is a membranous marker of Schwann cells and growth associated protein 43 (GAP43) is one of the most activated protein when nerve regeneration in the growth cone, which are usually used to evaluate efficacy of nerve regeneration [36, 37]. The harvested PPN samples were homogenized in RIPA tissue lysis buffer (50mM Tris pH 7.4, 150mM NaCl, 1% TritonX-100, 1% sodium deoxycholate, 0.1% SDS, 2mM sodium pyrophosphate, 25mM β-glycerophosphate) and centrifuged at 12,000g for 5min at 4°C. The protein concentration in the supernatant was measured by Master Kit with Split Buffer (Wes, ProteinSimple, USA); the supernatant was boiled for 10 min., thencentrifuged at 12,000g for 1 min. A total of 4 μg each was loaded on sample plate in a Simon system for protein separation at 375 V for 25 min. The transferred membranes were blocked separately with S100β (1:30, ab52642, abcam, Shanghai, China) and GAP43 (1:30, ab12274, abcam, Shanghai, China) antibodies for 15 min, incubated with rabbit anti S100β and GAP43, separately for 30 min and incubation with anti-rabbit secondary antibody IgG for 30 min at room temperature. The relative expression of axonal regrowth associated protein to β-actin was calculated and analyzed by Simple Western assays.

### Statistical analysis

All the data were presented as the mean ± standard deviation. Neuron regeneration data was evaluated by using receiver operating characteristic (ROC, SigmaPlot version 10.0.1, Systat software, USA). Comparisons among the groups were made by one-way ANOVA using SPSS 18.0. Post hoc test was done with Bonferroni correction for multiple comparisons. P < 0.05 was considered to be statistically significant.

## CONCLUSIONS

The nerve regeneration and bladder function recovery were effectively enhanced by FK1706. For neurogenic motor dysfunction caused by nerve injury and neurodegenerative diseases, FK1706 shall have a potential therapeutic value in the clinic. It has the unique advantages over traditional immunophilin ligands.
